# Metabolic Engineering of *Escherichia coli* for Producing Astaxanthin as the Predominant Carotenoid

**DOI:** 10.3390/md15100296

**Published:** 2017-09-22

**Authors:** Qian Lu, Yi-Fan Bu, Jian-Zhong Liu

**Affiliations:** Institute of Synthetic Biology, Biomedical Center, Guangdong Province Key Laboratory for Aquatic Economic Animals and South China Sea Bio-Resource Exploitation, School of Life Sciences, Sun Yat-sen University, Guangzhou 510275, China; luqian5@mail2.sysu.edu.cn (Q.L.); lssljz@outlook.com (Y.-F.B.)

**Keywords:** astaxanthin, *Escherichia coli*, metabolic engineering, β-carotene ketolase, β-carotene hydroxylase

## Abstract

Astaxanthin is a carotenoid of significant commercial value due to its superior antioxidant potential and wide applications in the aquaculture, food, cosmetic and pharmaceutical industries. A higher ratio of astaxanthin to the total carotenoids is required for efficient astaxanthin production. β-Carotene ketolase and hydroxylase play important roles in astaxanthin production. We first compared the conversion efficiency to astaxanthin in several β-carotene ketolases from *Brevundimonas* sp. SD212, *Sphingomonas* sp. DC18, *Paracoccus* sp. PC1, *P.* sp. N81106 and *Chlamydomonas reinhardtii* with the recombinant *Escherichia coli* cells that synthesize zeaxanthin due to the presence of the *Pantoea ananatis crtEBIYZ.* The *B.* sp. SD212 *crtW* and *P. ananatis crtZ* genes are the best combination for astaxanthin production. After balancing the activities of β-carotene ketolase and hydroxylase, an *E. coli* ASTA-1 that carries neither a plasmid nor an antibiotic marker was constructed to produce astaxanthin as the predominant carotenoid (96.6%) with a specific content of 7.4 ± 0.3 mg/g DCW without an addition of inducer.

## 1. Introduction

Astaxanthin is a carotenoid of significant commercial value due to its superior antioxidative, anti-inflammatory and anticancer features [1]. It has wide applications in the aquaculture, food, cosmetic and pharmaceutical industries. Currently, commercial astaxanthin is mainly synthesized chemically or extracted from natural producers such as the green algae *Haematococcus pluvialis* or the red yeast *Xanthophyllomyces dendrorhous*. Considering the limited productivity of astaxanthin via extraction and the biosafety issues of chemical synthesis, microbial production of astaxanthin via metabolic engineering has become an attractive alternative [2,3].

**Figure 1:**
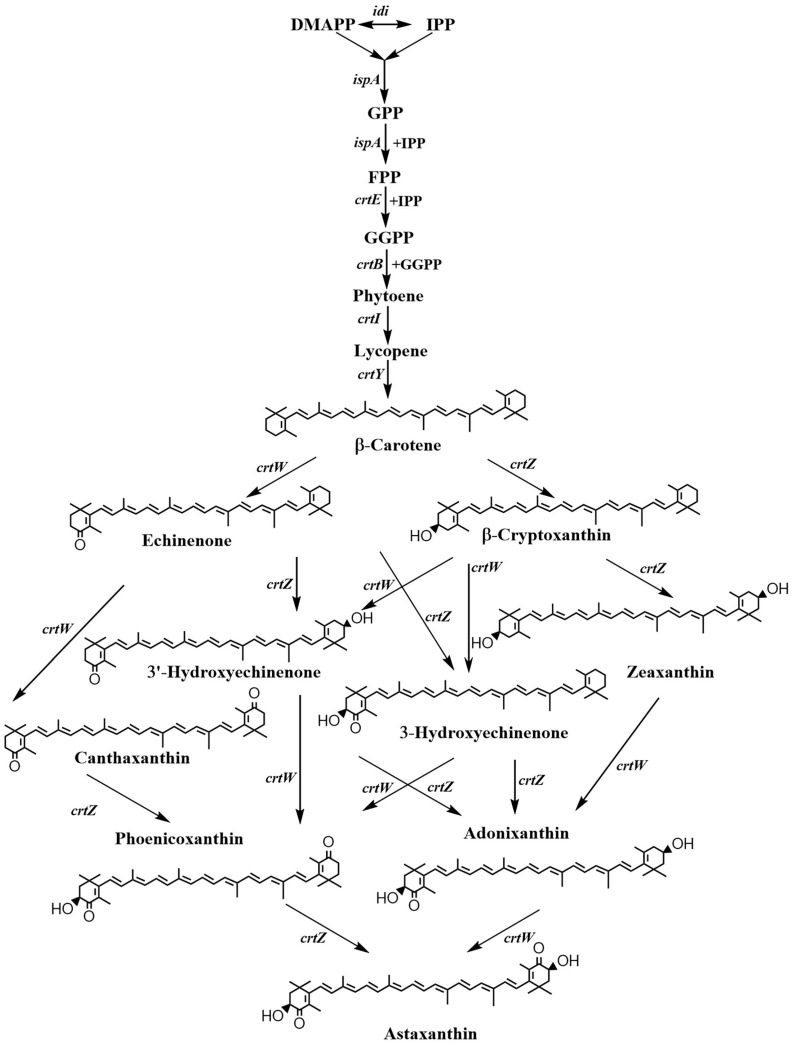
Pathway for biosynthesizing astaxanthin. DMAPP: Dimethylallyl diphosphate; GPP: Geranyl pyrophosphate; FPP: Farnesyl pyrophosphate; GGPP: Geranylgeranyl pyrophosphate; *ispA*: FPP synthase gene; *crtE*: GGPP synthase gene; *crtB*: Phytoene synthase gene; *crtI:* Carotene desaturase gene; *crtY*: Lycopene β-cyclase gene; *crtZ*: β-carotene hydroxylase gene; *crtW*: β-carotene ketolase gene.

In recent years, *Escherichia coli* [4], *Saccharomyces cerevisiae* [5,6] and *Corynebacterium glutamicum* [7] have been used as a host strain for astaxanthin production by the introduction of the astaxanthin biosynthesis pathway (Figure 1) into these non-carotenogenic microorganisms. Metabolic engineering *E. coli* for astaxanthin production has been widely reported in recent years. It has been demonstrated that the pathway from β-carotene to astaxanthin is a crucial step in astaxanthin synthesis [8]. The pathway requires two enzymes, β-carotene ketolase CrtW and β-carotene hydroxylase CrtZ. It has been shown that many bacterial CrtWs and CrtZs are bifunctional, with respect to their substrate [9,10]. They can accept β-carotene as well as its hydroxylated or ketolated products as a substrate, resulting in the formation of eight carotenoid intermediates which affect astaxanthin conversion as measured by the percentage of astaxanthin produced relative to the total carotenoid content (Figure 1). The astaxanthin ratio affects the production costs. To increase the astaxanthin ratio, many bacterial CrtWs and CrtZs have been identified and characterized [4,8,11,12,13,14,15,16,17]. However, the ratio reported in the above papers was lower than 90%. Thus, to increase the astaxanthin ratio, we first compared the conversion efficiency to astaxanthin in several CrtWs, which had a higher efficiency for astaxanthin production reported in the literature, with recombinant *E. coli* cells that synthesize zeaxanthin. Then, balancing the expressions of the two enzymes was carried out to obtain a plasmid-free *E. coli*, which produced astaxanthin of 7.4 ± 0.3 mg/g dry cell weight (DCW) with the astaxanthin ratio of 96.6% without the addition of an inducer.

## 2. Results and Discussion

### 2.1. Screening of β-Carotene Ketolase

It has been demonstrated that astaxanthin biosynthesis proceeds from β-carotene through hydroxylation by CrtZ and then ketolation by CrtW [14]. Moreover, our previous study has proven that *Pantoea ananatis crtZ* is superior to that of *P. agglomerans* or *H. pluvialis* for zeaxanthin production [18]. Thus, we first compared the catalytic efficiency for ketolating zeaxanthin to astaxanthin by different CrtWs. We selected four β-carotene ketolases with higher efficiencies for astaxanthin production reported in the literature as candidates. The four ketolases were *Brevundimonas* sp. SD212 CrtW [11,12], *Sphingomonas* sp. DC18 CrtW^F213L/R203W^ [8], *Paracoccus* sp. N81106 CrtW^L175W^ [16] and *Chlamydomonas reinhardtii* β-carotene ketolase (Bkt) [17]. The plasmids containing the β-carotene ketolase gene were transferred into an engineered zeaxanthin-producing strain *E. coli* ZEAX [19]. One copy of *P. ananatis crtZ* under the control of the P37 promoter was integrated into the chromosome of the β-Carotene producing strain *E. coli* BETA-1 [18]. Table 1 presents the results of astaxanthin production by the different engineered *E. coli.* Among the four β-carotene ketolase genes, the strain harboring *B.* sp. SD212 *crtW* produced a higher level of astaxanthin (2.7 ± 0.1 mg/g DCW), indicating that *B.* sp. SD212 *crtW* and *P. ananatis crtZ* genes are the best combinations for astaxanthin production. Misawa’s group also demonstrated that *B.* sp. SD212 *crtW* and *P. ananatis crtZ* genes are a combination of the most promising gene candidates for astaxanthin production [10,11,12]. Then we assembled two genes into one plasmid to increase the dose of the gene using BglBrick assembly technology and investigated its effect on the combination of different genes on astaxanthin production. As shown in Table 1, increasing the dose of the gene indeed enhanced astaxanthin production. *E. coli* ZEAX (pZS-2*crtW_Bsp_*) produced 4.6 ± 0.1 mg/g DCW of astaxanthin.

It has been shown that ketolase activity on zeaxanthin is the limiting step of astaxanthin biosynthesis in a bacterial and plant system [4,17]. To increase the astaxanthin ratio and produce efficiency, many bacterial CrtWs have been characterized and compared. It has been reported that the CrtW enzyme from *B.* sp. SD212 had a higher efficiency for converting zeaxanthin to astaxanthin than that from *P.* sp. PC1 and *P.* sp. N81106 [11]. Of the three β-carotene ketolase enzymes from *H. pluvialis*, *Chlorella zofingien* and *C. reinhardtii*, *C. reinhardtii* β-carotene ketolase had the highest activity for the conversion of zeaxanthin to astaxanthin [17]. Among *Rhodococcus erythropolis* PR4 CrtO, *Synechosistis* sp. PCC6803 CrtO and *B.* sp. SD212 CrtW, only *B.* sp. SD212 CrtW could synthesize astaxanthin from zeaxanthin [12]. Comparative analysis of the CrtO and CrtW revealed that CrtW was more efficient for the conversion of carotene to canthaxanthin than CrtO [14]. The conversion efficiency of *Gloeobacter violaceus* PCC 7421, *Anabaena* (also known as *Nostoc*) sp. PCC 7120 and *Nostoc punctiforme* PCC 73102 CrtW was compared in engineered *E. coli* [13]. The results demonstrated that the CrtW from *A.* sp. PCC 7120 as well as *N. punctiforme* PCC 73102 (CrtW148) can convert not only β-carotene but also zeaxanthin into canthaxanthin and astaxanthin, respectively [13].

Protein engineering of CrtW has been successfully used to improve astaxanthin production in recombinant *E. coli* cells that synthesize zeaxanthin. To improve *S.* sp. DC18 CrtW activity in hydroxylated carotenoids for astaxanthin production, *S.* sp. DC18 CrtW was evolved to obtain the R203W/F213L double mutant that yielded the highest improvement for astaxanthin production [8]. The strain harboring the double mutant produced astaxanthin as the predominant carotenoid (88%) [8]. By using random mutagenesis, *P.* sp. N81106 *crtW* mutants were generated [16]. The zeaxanthin producer *E. coli* harboring the *crtW*^L175^ mutant produced 78% of astaxanthin in the total carotenoid [16].

### 2.2. Balancing the Activities of β-Carotene Ketolase and Hydroxylase

**Figure 2:**
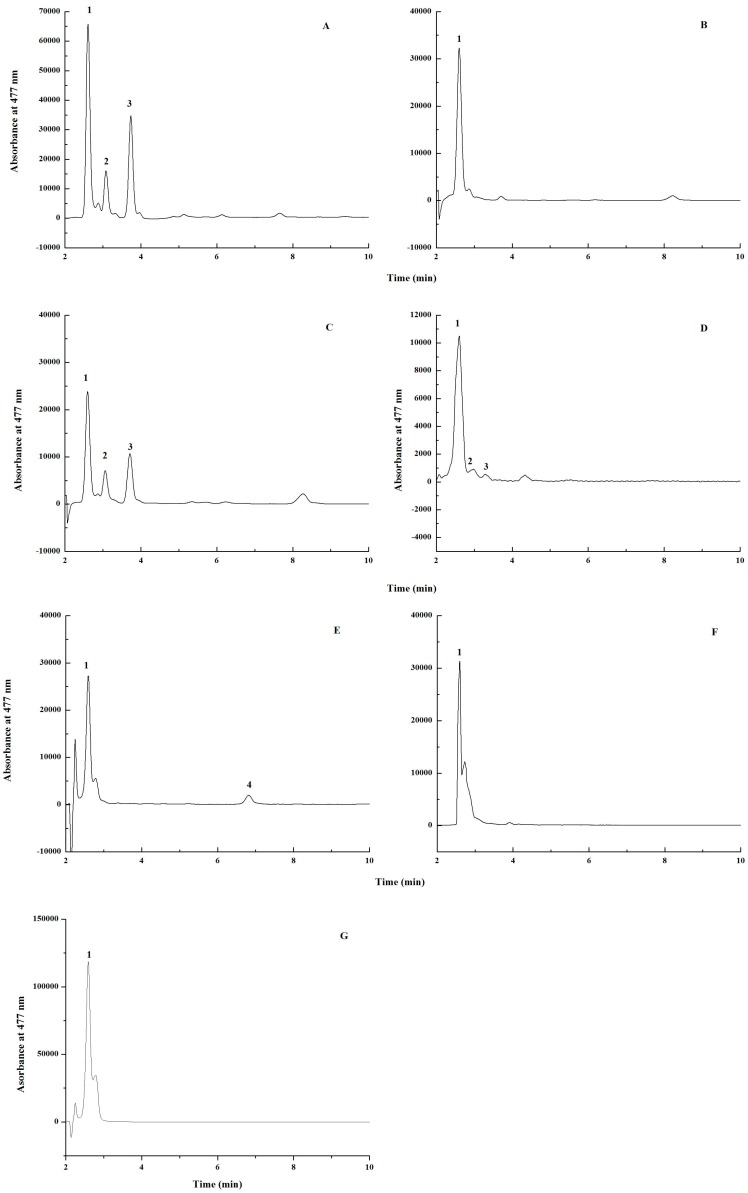
HPLC analysis of carotenoid products extracted from *E. coli* ZEAX (pZS-2*crtW_Bsp_*) (**A**), *E. coli* ZEAX (pZS-2*crtW_Bsp_*, pBAD-*crt*Z) (**B**), *E. coli* ZEAX (pZS-2*crtW_Bsp_*, pBAD-*crtW_Bsp_*) (**C**), *E. coli* ZEAX-4 (pZS-2*crtW_Bsp_*) (**D**), *E. coli* ASTA (**E**), *E. coli* ASTA-1 (**F**) and standard astaxanthin (**G**). 1. astaxanthin; 2. Phoenicoxanthin; 3. Canthaxanthin; 4. Lycopene.

We analyzed the accumulated carotenoids in *E. coli* ZEAX (pZS-2*crtW_Bsp_*) as shown in Figure 2A. The engineered strain produced 51.9% astaxanthin, 13.4% phoenicoxanthin and 30.4% canthaxanthin. From the biosynthetic pathway as shown in Figure 1, canthaxanthin and phoenicoxanthin are the intermediates of the pathway through first ketolation and then hydroxylation. Their accumulation indicates that the expression level of the hydroxylase gene *crtZ* may be low in this strain. Thus, we expressed pBAD-*crt*Z in *E. coli* ZEAX (pZS-2*crtW_Bsp_*) to verify our hypothesis. As shown in Figure 2B, co-expressing pBAD-*crt*Z with pZS-2*crtW_Bsp_* in *E. coli* ZEAX indeed increased the astaxanthin ratio to the total carotenoid content from 51.9% to 87.5%. We also co-overexpressed pBAD-*crt*W_Bsp_ with pZS-2*crtW_Bsp_* in *E. coli* ZEAX. As shown in Figure 2C, the co-overexpression decreased the astaxanthin ratio from 51.9% to 46.4% and increased the canthaxanthin and phoenicoxanthin ratio. This stands in contrast to the study by Lemuth et al. [4], who found that increasing the ketolase activity or decreasing the hydroxylase activity would be necessary for astaxanthin production. Our results demonstrated that increasing CrtZ activity would be necessary for producing astaxanthin as the predominant carotenoid. Thus, to increase the expression level of *crtZ*, we integrated two copies of the *crtZ* into the chromosome of the β-carotene producer *E. coli* BETA to generate the zeaxanthin producer *E. coli* ZEAX-4. The recombinant *E. coli* ZEAX-4 harboring pZS-2*crtW_Bsp_* produced 88.6% astaxanthin, 3.9% phoenicoxanthin and 3.0% canthaxanthin (Figure 2D).

To reduce the metabolic burden and to avoid antibiotic markers resulting from the plasmid, we integrated *B.* sp. SD212 *crtW* into the chromosome of the zeaxanthin producer *E. coli* ZEAX-4 to generate an astaxanthin producer *E. coli* ASTA. The resulting strain *E. coli* ASTA produced 92.6% astaxanthin (Figure 2E). However, lycopene was also detected in *E. coli* ASTA. We guess the phenomenon may be due to the lower expression level of the *crtY*. Thus, we integrated another copy of the *crtY* into the chromosome of *E. coli* ASTA to obtain *E. coli* ASTA-1. This integration enhanced the astaxanthin ratio to 96.6% (Figure 2F). *E. coli* ASTA-1 produced astaxanthin as the predominant carotenoid (96.6%) with a specific content of 7.4 ± 0.3 mg/g DCW (Figure 2F).

It is supposed that β-carotene hydroxylase and ketolase compete for their substrate and that only a balanced expression of these two enzymes might result in a complete conversion of β-carotene to astaxanthin [4,14,20,21]. To allow a variable expression of *crtZ* compared to the tac-promoter controlled *N. punctiforme* PCC 73102 *crtW148* and *P. ananatis crtEBIY*, *P. ananatis crtZ* was expressed under the control of the rhamnose-promoter [4]. The engineered strain *E. coli* BW-ASTA produced astaxanthin as the predominant carotenoid (95%) at a concentration of 1.4 mg/g DCW in minimal medium with glucose and Isopropyl β-d-thiogalactoside (IPTG) [4]. The *E. coli* strain with the pTrc*CrtW-*pBAD*CrtZ* dual expression systems had an increased selectivity for astaxanthin production (1.99 mg/g DCW, about 90%) [14]. Our study also suggests that appropriate activities of β-carotene hydroxylase and ketolase are important for astaxanthin production.

Astaxanthin production by microorganisms is summarized in Table 2. Although Lemuth et al. first engineered a plasmid-free *E. coli* strain for astaxanthin production, the strain produced astaxanthin of 1.4 mg/g DCW with an astaxanthin ratio of 95% only with IPTG induction [4]. In this study, we engineered a plasmid-free *E. coli* for astaxanthin production, which reached 7.4 ± 0.3 mg/g DCW with the astaxanthin ratio of 96.6% without the addition of an inducer. From Table 2, we can see that the astaxanthin ratio obtained in this study is the highest value. However, the astaxanthin yield obtained in this study is slightly lower than that (8.64 mg/g DCW) in *E. coli* reported by Ma et al., which is the highest astaxanthin yield reported to date [22]. In their study, the upper mevalonate (MEV) pathway operon from *S. cerevisiae*, the lower MEV pathway operon from *S. cerevisiae*, plus *E. coli idi* and the optimized astaxanthin biosynthetic pathway genes were expressed on three different plasmids [22]. The optimized astaxanthin biosynthetic pathway genes contain *P. ananatis crtEBI* under the control of the *P_T_*_7_ promoter, *P. agglomerans crtY* and *crtZ, B. sp.* SD212 *crtW* and *E. coli idi* under the control of the *P_T_*_7_ promoter [22]. Thus, the introduction of an MEV pathway in our strain *E. coli* ASTA-1 may further increase astaxanthin production.

## 3. Materials and Methods

### 3.1. Strains, Plasmids and Primers

Strains, plasmids and primers used in this study were listed in Table 3.

### 3.2. Genetic Methods

After codon optimization for *E. coli* codon usage by using the 31C method reported by Boël et al. [25], *B.* sp. SD212 *crtW*, *S.* sp. DC18 *crtW^F213L/R203W^*, *P.* sp. N81106 *crtW^L175W^* and *C. reinhardtii bkt* genes were synthesized by Suzhou GENEWIZ, Inc. (Suzhou, China) and ligated into pUC57. The gene fragment was then digested and inserted into the *Nhe*I/*Kpn*I sites of pZSBP [18] to obtain pZS-*crt*W_BSP_, pZS-*crt*W_SSP_, pZS-*crt*W_PSP_ and pZS-*bkt*, respectively. The BglBrick standard assembling method was used to assemble the above any two genes into a plasmid.

### 3.3. Astaxanthin Production in Shake Flasks

A single colony was inoculated into 5 mL of Luria-Bertani (LB) medium supplemented with 5 g/L KAc in a falcon tube which was incubated overnight at 37 °C. The overnight seed culture was then inoculated into 50 mL of the Super Broth with ammonium and sucrose (SBMSN) medium with an initial OD_600_ of 0.1. The SBMSN medium (pH 7.0) contained 5 g/L sucrose, 12 g/L peptone, 24 g/L yeast extract, 1.7 g/L KH_2_PO_4_, 11.42 g/L K_2_HPO_4_, 1 g/L MgCl_2_·6H_2_O, 1.42 g/L ammonium oxalate, and 2 g/L Tween-80. The cultures were incubated at 37 °C for 48 h in a rotary shaking incubator set to 150 rpm. Cell growth was measured according to the OD_600_ and converted into DCW (g/L) using a standard curve.

### 3.4. Extraction and Quantification of Carotenoids

Cells were extracted with acetone to isolate carotenoids as described previously [9]. *E. coli* cultures (250 μL) were harvested by centrifugation at 12,000 rpm for 5 min. The cell pellet was washed with water and extracted with 1 mL of acetone at 55 °C for 15 min with intermittent vortexing. The acetone supernatant after centrifugation was transferred to a new tube. Carotenoids were analyzed by HPLC (Shimadzu HPLC system, Model LC-20A, Shimadzu, Japan) using an Inertsil ODS-SP column (5 μm, 4.6 × 150 mm, GL Sciences Inc., Tokyo, Japan). The mobile phase of acetonitrile-methanol (65:35 *v*/*v*) at a flow rate of 1 mL/min was used. The absorbance of carotenoids at 477 nm was detected using a photodiode array detector (SPD-M20A). Carotenoid compounds were identified on the basis of their retention times relative to standard compounds (Sigma-Aldrich, St. Louis, MO, USA). Astaxanthin was quantified by comparing the integrated peak areas with that of authentic standards. The contents of total carotenoids were approximated via application of the astaxanthin curve.

### 3.5. Statistical Analysis

All experiments were performed in triplicate, and the data are presented as the mean of the three experiments ± standard deviation. Tukey’s test was carried out for the statistical analysis using the OriginPro (version 7.5) package. Statistical significance was defined as *p* < 0.05.

## Figures and Tables

**Table 1:** Effect of the overexpression of different β-carotene ketolase genes on astaxanthin production in *Escherichia coli* ZEAX.

Plasmid	OD_600_ *	Astaxanthin Concentration, mg/L	Astaxanthin Content, mg/gDCW
Single gene			
pZS-*crtW_Bsp_*	9.55 ± 0.16	8.1 ± 0.1	2.7 ± 0.1
pZS-*crtW_Psp_*	8.05 ± 0.47	1.3 ± 0.1	0.5 ± 0.1
pZS-*crtW_Ssp_*	8.61 ± 0.21	0.8 ± 0.1	0.3 ± 0.1
pZS-*bkt*	11.07 ± 0.20	5.0 ± 0.2	1.4 ± 0.1
Double genes			
pZS-2*crtW_Bsp_*	19.67 ± 0.33	28.8 ± 0.2	4.6 ± 0.1
pZS-2*bkt*	22.15 ± 0.33	20.9 ± 1.6	3.5 ± 0.1
pZS-2*crtW_Psp_*	16.82 ± 0.56	7.0 ± 0.1	1.3 ± 0.1
pZS-2*crtW_Ssp_*	14.91 ± 0.31	1.1 ± 0.6	0.2 ± 0.1
Mixed genes			
pZS-*crtW_Bsp_crtW_Psp_*	19.93 ± 0.38	12.6 ± 0.6	2.0 ± 0.1
pZS-*crtW_Bsp_crtW_Ssp_*	21.73 ± 0.19	24.6 ± 0.4	3.5 ± 0.1
pZS-*crtW_Bsp_bkt*	19.9 ± 1.27	20.9 ± 1.6	3.3 ± 0.1
pZS-*crtW_Psp_crtW_Ssp_*	16.6 ± 0.17	8.5 ± 0.2	1.6 ± 0.2
pZS-*crtW_Psp_bkt*	20.01 ± 0.12	10.5 ± 1.6	1.6 ± 0.1
pZS-*crtW_Ssp_bkt*	21.57 ± 0.38	11.2 ± 0.7	1.6 ± 0.1

* The OD_600_ value was expressed as cell growth.

**Table 2:** Astaxanthin production by different microorganisms.

Strain	Astaxanthin Yield	Astaxanthin Ratio (%)	Reference
*E. coli*	5.8 mg/g DCW	N.D. *	[23]
*E. coli*	8.64 mg/g DCW	N.D.	[22]
*E. coli*	1.4 mg/g DCW	95	[4]
*E. coli*	1.99 mg/g DCW	90	[14]
*E. coli*	7.4 ± 0.3 mg/g DCW	96.6	This study
*S. cerevisiae*	4.7 mg/g DCW	N.D.	[5]
*S. cerevisiae*	8.10 mg/g DCW	N.D.	[6]
*C. glutamicum*	0.4 mg/L/h	N.D.	[7]

* N.D. = not determined.

**Table 3:** Strains and plasmids used in this study.

Name	Description	Reference/Sources
Strain		
*E. coli* BETA-1	β-Carotene producing strain	[18]
*E. coli* ZEAX	Zeaxanthin producing strain, one copy of *Pantoea ananatis crtZ* under the control of the P37 promoter was integrated into *E. coli* BETA-1 chromosome	[19]
*E. coli* ZEAX-4	Zeaxanthin producing strain, two copies of *P. ananatis crtZ* under the control of the P37 promoter was integrated into *E. coli* BETA-1 chromosome	This study
*E. coli* ASTA	Astaxanthin producer, one of *B.* sp. SD212 *crtW* under the control of the P37 promoter was integrated into *E. coli* ZEAX-4 chromosome	This study
*E. coli* ASTA-1	Astaxanthin producer, another copy of *P. ananatis crtY* under the control of the P37 promoter was integrated into *E. coli* ASTA-1 chromosome	This study
Plasmid		
pZSABP	Constitute expression vector, pSC101 *ori*, P37 promoter, Amp^r^, BglBrick, ePathBrick containing four isocaudamer (*Avr*II, *Nhe*I, *Spe*I and *Xba*I)	[18]
pBAD33	Expression vector, P_BAD_, p15A *ori*, Cm^r^	[24]
pZS-*crtW_Bsp_*	pZSABP containing *Brevundimonas* sp. SD212 *crtW* under the control of the P37 promoter	This study
pZS-*crtW_Psp_*	pZSABP containing *Paracoccus* sp. N81106 *crtW^L175W^* under the control of the P37 promoter	This study
pZS-*crtW_Ssp_*	pZSABP containing *Sphingomonas* sp. DC18 *crtW^F213L/R203W^* under the control of the P37 promoter	This study
pZS-*bkt*	pZSABP containing *Chlamydomonas reinhardtii bkt* under the control of the P37 promoter	This study
pZS-2*crtW_Bsp_*	pZSABP containing two copies of *B.s* sp. SD212 *crtW* under the control of the P37 promoter	This study
pZS-2*bkt*	pZSABP containing two copies of *C. reinhardtii bkt* under the control of the P37 promoter	This study
pZS-2*crtW_Psp_*	pZSABP containing two copies of *P.* sp. N81106 *crtW^L175W^* under the control of the P37 promoter	This study
pZS-2*crtW_Ssp_*	pZSABP containing two copies of *S.* sp. DC18 *crtW^F213L/R203W^* under the control of the P37 promoter	This study
pZS-*crtW_Bsp_crtW_Psp_*	pZSABP containing *B.s* sp. SD212 *crtW* under the control of the P37 promoter and *P.* sp. N81106 *crtW^L175W^* under the control of the P37 promoter	This study
pZS-*crtW_Bsp_crtW_Ssp_*	pZSABP containing *B.s* sp. SD212 *crtW* under the control of the P37 promoter and *S.* sp. DC18 *crtW^F213L/R203W^* under the control of the P37 promoter	This study
pZS-*crtW_Bsp_bkt*	pZSABP containing *B.s* sp. SD212 *crtW* under the control of the P37 promoter and *C. reinhardtii bkt* under the control of the P37 promoter	This study
pZS-*crtW_Psp_crtW_Ssp_*	pZSABP containing *P.* sp. N81106 *crtW^L175W^* under the control of the P37 promoter and *S.* sp. DC18 *crtW^F213L/R203W^* under the control of the P37 promoter	This study
pZS-*crtW_Psp_bkt*	pZSABP containing *P.* sp. N81106 *crtW^L175W^* under the control of the P37 promoter and *C. reinhardtii bkt* under the control of the P37 promoter	This study
pZS-*crtW_Ssp_bkt*	pZSABP containing *S.* sp. DC18 *crtW^F213L/R203W^* under the control of the P37 promoter and *C. reinhardtii bkt* under the control of the P37 promoter	This study
pBAD-*crt*Z	pBAD33 containing *P. ananatis crtZ*	This study
pBAD-*crt*W_Bsp_	pBAD33 containing *B.s* sp. SD212 *crtW*	This study

## Abbreviations

*B.* sp.	*Brevundimonas* sp.
*S.* sp.	*Sphingomonas* sp.
*P.* sp.	*Paracoccus* sp.
*P. ananatis*	*Pantoea ananatis*
*H. pluvialis*	*Haematococcus pluvialis*
*P. agglomerans*	*Pantoea agglomerans*
*C. reinhardtii*	*Chlamydomonas reinhardtii*
*A.* sp.	*Anabaena* sp.
*N. punctiforme*	*Nostoc punctiforme*
MEV	mevalonate
